# Exploring User Experiences of the Mom2B mHealth Research App During the Perinatal Period: Qualitative Study

**DOI:** 10.2196/53508

**Published:** 2024-08-08

**Authors:** Ayesha-Mae Bilal, Konstantina Pagoni, Stavros I Iliadis, Fotios C Papadopoulos, Alkistis Skalkidou, Caisa Öster

**Affiliations:** 1 Department of Medical Sciences Psychiatry Uppsala University Uppsala Sweden; 2 Centre for Women's Mental Health During the Reproductive Lifespan (WOMHER) Uppsala University Uppsala Sweden; 3 Department of Women's and Children's Health Uppsala University Uppsala Sweden

**Keywords:** digital phenotyping, smartphone app, mHealth, mobile health, qualitative study, user experience, usability, perinatal depression, depression, app, user, users, qualitative, perinatal, mobile app, clinical research, acceptability, behavioral data, depressive symptoms, interview, pregnant, postpartum, women, thematic analysis, well-being, monitor, mobile phone

## Abstract

**Background:**

Perinatal depression affects a significant number of women during pregnancy and after birth, and early identification is imperative for timely interventions and improved prognosis. Mobile apps offer the potential to overcome barriers to health care provision and facilitate clinical research. However, little is known about users’ perceptions and acceptability of these apps, particularly digital phenotyping and ecological momentary assessment apps, a relatively novel category of apps and approach to data collection. Understanding user’s concerns and the challenges they experience using the app will facilitate adoption and continued engagement.

**Objective:**

This qualitative study explores the experiences and attitudes of users of the Mom2B mobile health (mHealth) research app (Uppsala University) during the perinatal period. In particular, we aimed to determine the acceptability of the app and any concerns about providing data through a mobile app.

**Methods:**

Semistructured focus group interviews were conducted digitally in Swedish with 13 groups and a total of 41 participants. Participants had been active users of the Mom2B app for at least 6 weeks and included pregnant and postpartum women, both with and without depression symptomatology apparent in their last screening test. Interviews were recorded, transcribed verbatim, translated to English, and evaluated using inductive thematic analysis.

**Results:**

Four themes were elicited: acceptability of sharing data, motivators and incentives, barriers to task completion, and user experience. Participants also gave suggestions for the improvement of features and user experience.

**Conclusions:**

The study findings suggest that app-based digital phenotyping is a feasible and acceptable method of conducting research and health care delivery among perinatal women. The Mom2B app was perceived as an efficient and practical tool that facilitates engagement in research as well as allows users to monitor their well-being and receive general and personalized information related to the perinatal period. However, this study also highlights the importance of trustworthiness, accessibility, and prompt technical issue resolution in the development of future research apps in cooperation with end users. The study contributes to the growing body of literature on the usability and acceptability of mobile apps for research and ecological momentary assessment and underscores the need for continued research in this area.

## Introduction

### Background

Perinatal depression (PND) impacts anywhere from 12% to 20% of women during pregnancy and after birth [1]. In Sweden, universal screening for PND takes place during a postpartum visit to the children’s health center and is done using the Edinburgh Postnatal Depression Scale (EPDS) [2]. Although efforts are being made to improve screening in the perinatal period, there are many barriers at both the patient and system level that prevent timely detection and intervention [3-5]. As such, early identification remains a challenge, with one Swedish study reporting that anywhere between 30% and 45% of women do not get screened, with some groups being at greater risk than others of being missed [6]. Early identification of individuals at risk of depression in the perinatal period is imperative for the implementation of timely and cost-effective interventions and improved prognosis [7].

Technological advancements in mobile health (mHealth) apps offer the opportunity to overcome barriers in health care provision. In 2019, over 90% of the population in Sweden owned a smartphone [8]. Their ubiquity and ability to unobtrusively amass large amounts of data in real time regarding the user’s functions and behaviors in their everyday life make them feasible tools to monitor mental health symptoms and identify users at risk of poor well-being.

Data collected can include both passive data from smartphone sensors, logs, and metadata as well as ecological momentary assessments [9], which are in situ, real-time data collection methods, such as app-based self-report scales. These data can be leveraged to develop social, behavioral, and cognitive phenotypes of individuals, which can subsequently be used to infer the user’s psychological state and other health indicators in a process termed “digital phenotyping” [10]. Smartphone-based digital phenotyping maintains the objectivity and the temporal and contextual integrity of diagnostically relevant information, as it overcomes the reliance on retrospective self-reporting from patients [9]. This enables the collection of rich, multivariable, and large-scale data sets that can be combined with advanced machine learning techniques to personalize health care, improve diagnostic validity, and predict disease and treatment outcomes [11].

Smartphone apps are being increasingly used as tools for digital phenotyping in psychiatry to support diagnosis and screening, as they enable the collection of data from both smartphone sensors and logs as well as subjective self-reports, cognitive tests, and other participation-based tasks. Recent studies have used the data collected from such digital phenotyping apps to apply machine learning methods to predict symptoms of mental illness, such as depression and anxiety [12-14], bipolar disorder [15,16], psychosis [17], and schizophrenia [18,19], and have focused on various vulnerable groups, such as veterans [20], students [21], as well as women in the perinatal period [22-25].

However, there are significant practical, social, and ethical challenges that impact users’ acceptance of and continued engagement with the app and raise concerns regarding privacy and data security [26,27]. Understanding the users’ diverse needs and priorities will enable us to alleviate these challenges and provide appropriate incentives. Exploring the issue of low engagement is especially relevant in populations that are experiencing depression, as its symptoms can diminish the influence of incentives [28]. These issues can lead to missing data, which can create biases in and reduce the accuracy of prediction models that can result from these data [17]. Although a few studies have investigated the feasibility and user experience of digital phenotyping apps [20,24,29], continued research is needed to explore user perspectives in more diverse populations and with more complex apps. Doing so will enable us to incorporate this knowledge in the initial stages of the app and study design process to enhance the acceptability and feasibility of such apps.

### Mom2B App

Mom2B (Uppsala University) is a smartphone app–based research study that aims to collect digital phenotyping data to ultimately develop and evaluate prediction models for PND [30]. The Mom2B app was developed as a means to collect digital phenotyping data from research participants. Data collected include active data, in the form of in-app self-report surveys and voice recording tasks, and passive data, that is, data collected from smartphone sensors and logs regarding the user’s mobility and sleep patterns, internet and smartphone use, and social media activity. Privacy protection measures are put in place to ensure that GPS data only concern relative movement, not actual location, and social media data only include the frequency of activity, not actual content. Depression symptoms are assessed as the outcome measure using the EPDS at various time points throughout the perinatal period. The array of data collected from this app is subsequently used to develop and evaluate prediction models for PND [31]. The models are not used within the app itself but can be evaluated in clinical settings in future studies.

**Figure 1:**
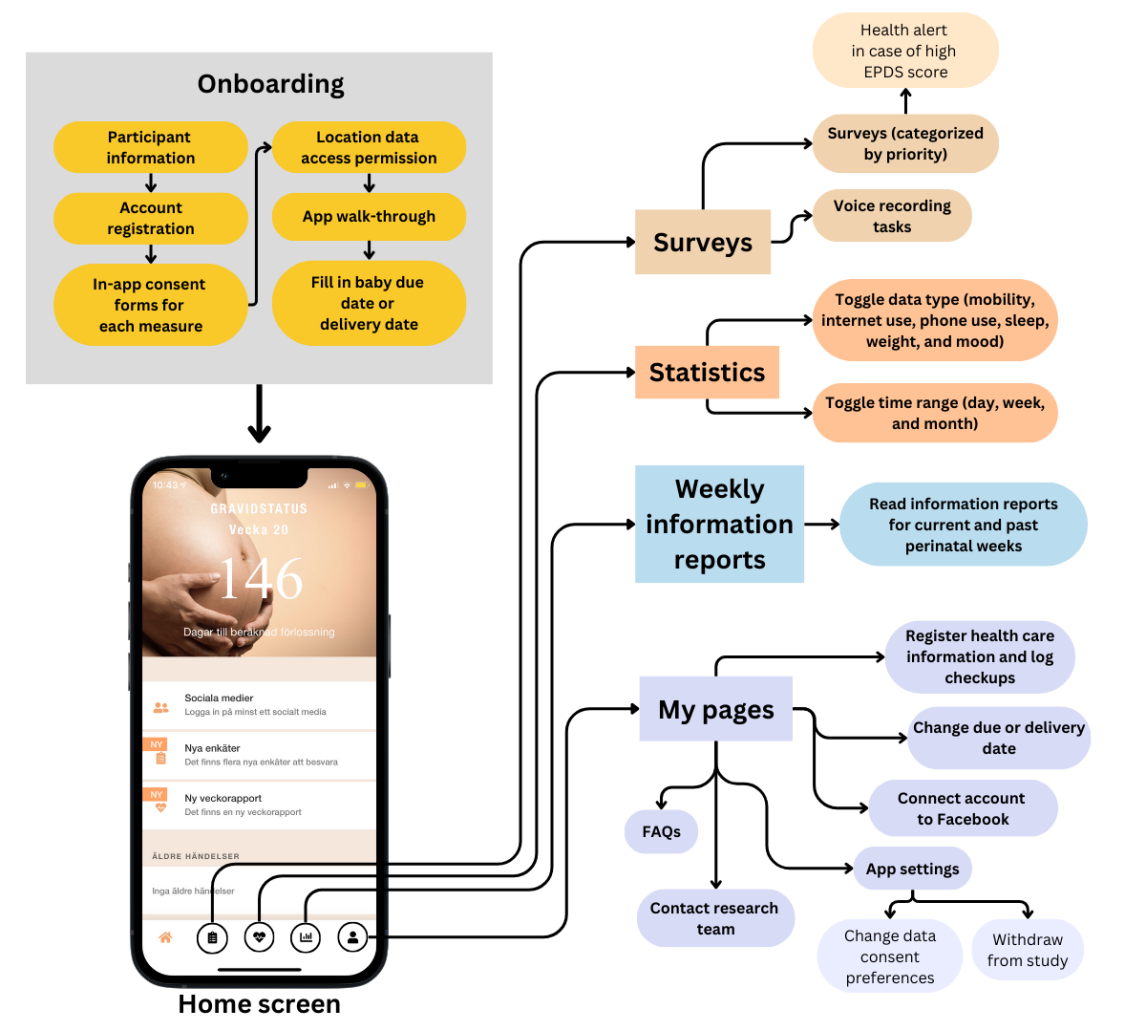
The onboarding process involves users being presented with information about the study and being asked to provide consent to the various types of data. Users are then asked to set their expected or actual delivery date, and the app configures its features according to the perinatal week they are calculated to be in. In the home screen, users can access the app’s various features. “Surveys” are set to be available for limited time periods and include voice recording tasks and the EPDS. A score over 12 in the EPDS prompts a message informing the user of their high score and the resources they can access to seek support and help. “Weekly reports” include information taken from 1177.se (Swedish national health care) to give week-by-week information snippets related to the pregnancy or newborn infant. Finally, “Statistics” allows users to view their activity and well-being over various periods of time based on their data. EPDS: Edinburgh Postnatal Depression Scale; FAQ: frequently asked question.

Participants can enable or disable any type of data from being collected as part of their consent preferences at any point in the study. Apart from the surveys and voice recording tasks, the main interactive features of the app are the weekly information reports and the statistical graphs. More information about these features as well as an overview of the main pages, content, and features of the Mom2B are presented in Figure 1.

### Objectives

This study aimed to explore the experiences and attitudes of Mom2B app users during the perinatal period. We particularly sought to investigate the acceptability of the app and participants’ concerns about providing data in this way.

## Methods

### Study Design

To explore users’ experiences with and attitudes toward using the Mom2B app, a qualitative focus group study was conducted. Focus group interviews were chosen to gather a larger amount of information with limited time resources [32]. We used inductive thematic analysis as described by Braun and Clarke [33] to analyze the data. The 32-item COREQ (Consolidated Criteria for Reporting Qualitative Studies) checklist [34] was followed as a guide to ensure the quality of reporting.

### Ethical Considerations

The study was approved by the Swedish Ethical Review Authority (2020/06645), and all participants provided informed consent. Participants were provided with participant information, where they were informed about their right to withdraw from the study at any time. The option to withdraw anytime was emphasized again at the start of the interviews. All interviews were kept confidential, with transcripts pseudoanonymized to remove any identifiable details. Additionally, to safeguard against potential identification, interview transcripts were not uploaded to public data repositories, and the data remained within the research team. Participants did not receive any compensation for their involvement.

### Participants

Users of the Mom2B app were recruited from the existing Mom2B cohort between December 2021 and May 2022. Participants had to have been active users of the app for at least 6 weeks in the perinatal period they were recruited to representative and must have completed at least 1 of their last 3 EPDS surveys. Women who had not updated their delivery date post partum, withdrawn from the study, or not consented to being contacted for participation in substudies (like this one) were excluded. To ensure a representative participant group and capture perspectives on the full scope of the app, including features only available to women exhibiting depression symptoms or to women in the pregnancy or postpartum period, we elected to use purposive random sampling. We stratified the cohort into 4 categories based on participants’ perinatal status (pregnant or postpartum at the time of recruitment) and whether or not they reported recently experiencing depression symptoms (women were considered depressed if their latest EPDS score on the app was 12 or above and considered as not depressed if the score was 10 or below).

We aimed for focus groups of 5 to 6 participants, with an equal distribution of women from all 4 stratification categories in each focus group. In total, 65 women consented to participate in the study; however, 24 dropped out before the interview, most often because of reasons related to their newborn infant. We continued to recruit participants to new focus groups until we agreed that information saturation had been reached [35].

A total of 41 participants were interviewed in the form of 13 focus groups, ranging in size from 2 to 5, and 1 was interviewed individually due to other expected participants in that group dropping out. Participants’ duration of app use ranged from 16 to 130 weeks. Additional participant characteristics are detailed in Tables 1 and 2.

**Table 1:** Participant demographics and characteristics (N=41).

Characteristics			Values, n (%)
**Age group (years)**			
	18-29	3 (7)	
	30-34	20 (49)	
	35-45	18 (44)	
**Country of origin**			
	Sweden	40 (98)	
	Other Nordic country	1 (2)	
**Education**			
	Postsecondary education	35 (85)	
	Secondary or lower	5 (12)	
	Unknown	1 (2)	
**Employment status**			
	Full-time	24 (59)	
	Part-time	3 (7)	
	Student	2 (5)	
	Unknown	12 (29)	
**Number of pregnancies**			
	Primigravida	18 (44)	
	Multigravida	21 (51)	
	Unknown	2 (5)	

**Table 2:** Distribution of participants (N=41) stratified by their perinatal status and depression symptomology to ensure a representative sample of the cohort.

Stratification categories		Values, n (%)		EPDS^a^ score	
				Mean (SD)	Range
**Pregnant**					
	Depressive symptoms present	7 (17)	13.8 (2.1)		12-17
	No depressive symptoms	6 (15)	3 (2.3)		1-7
**Post partum**					
	Depressive symptoms present	7 (17)	14.1 (2)		12-18
	No depressive symptoms	21 (51)	3.8 (2.4)		0-8

^a^EPDS: Edinburgh Postnatal Depression Scale.

### Procedure

Participants were recruited via email invitation and signed consent forms digitally. They were then able to select the focus group that best suited their availability and were sent 2 reminder emails before the interview with a brief of the interview topics to allow participants to have some time to reflect and gather their thoughts before the interview. The first author (AMB, female) served as a moderator, recorded the session, and took notes, while the second author (KP, female) conducted the interviews in Swedish over video conference.

### Data Collection

An interview guide (Multimedia Appendix 1) with semistructured questions was developed by the research team and used to prompt topics of discussion in the focus group. The first 2 focus group interviews were considered pretests to allow the research team to make any necessary revisions. Based on these interviews, minor changes were made to the recruitment procedures and the wording of some questions in the interview guide to improve clarity. The pretests were judged as contributing relevant data and were included in the analysis. The participants were first given background information on the Mom2B app–based research study and an overview of the purpose of this interview study. Finally, participants were debriefed about how their answers would be used and where to reach out with questions or concerns. Interviews lasted for 20-50 minutes and were recorded, and the audios were submitted for transcription.

### Data Analysis

We carefully considered the research question and its focus on capturing the voices and perspectives of participants, as well as the relative novelty of user experience research with digital phenotyping apps, and deemed inductive thematic analysis to be the most appropriate approach. We analyzed the English-translated transcripts following Braun and Clarke’s model for reflexive thematic analysis based on the model “Codebook” analysis [33,36]. The analysis was performed in NVivo (version 13; Lumivero) by 3 of the authors.

The first author (AMB) thoroughly read and reread all transcripts to familiarize with the data and then systematically analyzed the data to generate initial codes using an open, semantic approach, as we were interested in exploring users’ stated opinions and impressions. The first and last author (AMB and CÖ) frequently discussed their different perspectives and revised codes in an iterative process as transcripts continued to be coded, as well as after coding was completed.

Preliminary themes were then generated based on meaningful patterns emerging within the coded data. Themes were initially largely categorical to allow for more intuitive sorting, and many codes were sorted into more than 1 theme at this point. The second author (KP) independently evaluated the codes to assess their validity and identify themes, and then all 3 authors (AMB, KP, and CÖ) came together to discuss how the codes can be refined and to further develop the themes so that they are sufficiently unique, make sense in the context of the data set, and truly reflect the cruces of the focus group discussions. Finally, themes and subthemes were defined in an iterative process.

## Results

### Overview

**Figure 2:**
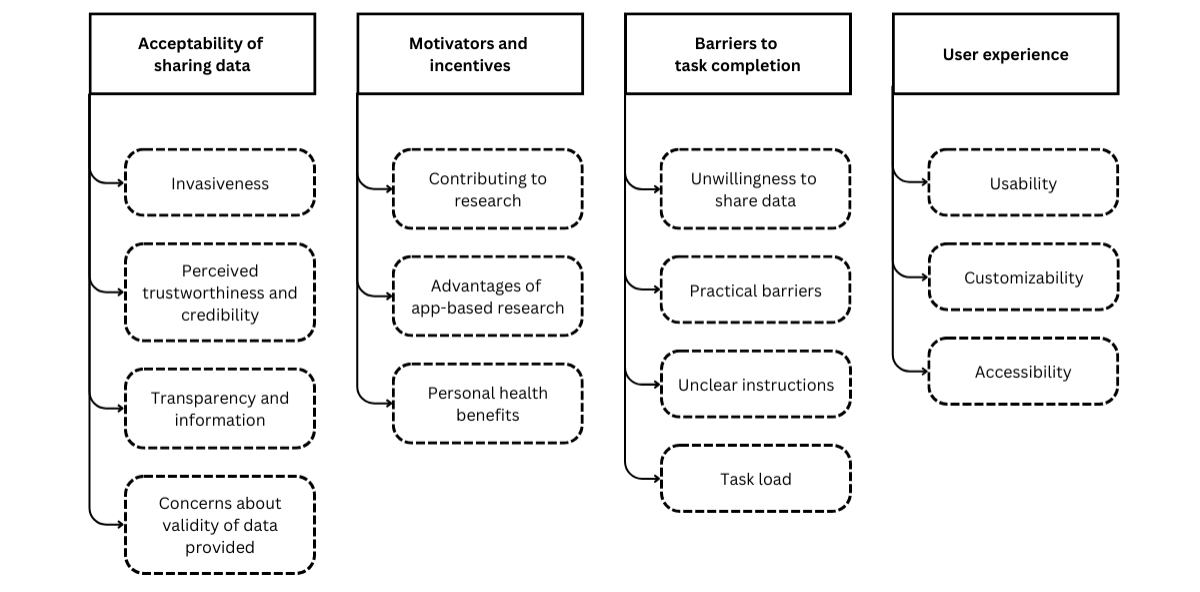
Visualization of the overarching themes and associated subthemes from the thematic analysis of the data.

In total, 4 themes were identified and are described below (in no particular order) with subthemes and supporting quotations. Figure 2 gives an overview of the identified themes and subthemes.

### Acceptability of Sharing Data

Given the large amount of data (with much of it being sensitive health and personal information) being collected, users’ perceptions on sharing that data are an important consideration.

#### Invasiveness

Participants had mixed opinions about all the different types of data they had to consent to. Some reported initially feeling uncomfortable about the idea of the app accessing their social media or GPS data or giving access to their medical records. Others felt that the data collected was reasonable, considering that they were part of a research study. However, participants were reassured by their control over what data they chose to share as well as the understanding that social media and GPS data tracked activity and mobility, as opposed to content and location.

It would have been a deal-breaker...[but] it doesn’t keep track of what I write in social media, but just that I interact, how much I, for example, liked something...

Participant 41, focus group 14

#### Perceived Trustworthiness and Credibility

The app being affiliated with Uppsala University and the data being collected for research purposes and being handled by researchers were important mitigating factors for users’ willingness to share sensitive and personal data. Participants trusted how their data are stored and used as well as the information they get from the app.

I wouldn’t have agreed to [the consent forms] if it wasn’t a research study, or that it wasn’t from a university or the healthcare system or something. I wouldn’t have agreed to this if it was the private sector. That made me also trust that [my data] was handled correctly...

Participant 41, focus group 14

#### Transparency and Information

Closely related to participant’s perceptions of invasiveness and trust was their expressed desire to know more about why that data were collected and what they had been used for. It was more so a matter of curiosity than concern; however, it may still impact their motivation to submit data.

These audio recordings, for example, in what way can it be used in research? I’m a little curious about how you can use it.

Participant 5, focus group 1

I would have liked to see even more information about it, like, how to use the results and how it can benefit others.

Participant 27, focus group 9

In some cases, the information participants wanted was, in fact, available in the participant information and consent forms; however, it appeared that users’ perceptions of trust in the study had led them to skim through these forms and miss relevant information.

I didn’t read everything in detail, but kind of felt that in a study conducted by a serious group, I trust that the information will be used in a way that is safe for me...I probably skimmed most of it.

Participant 2, focus group 1

#### Concerns About Validity of Data Provided

Participants expressed concern about whether the data they provided, particularly on mood-related surveys, accurately reflected the truth. Many participants felt that poor scores on mood surveys were more reflective of the effects of social isolation during the COVID-19 pandemic, as well as preexisting mental health conditions, rather than being pregnant or having given birth.

For me, my mood was more based on the fact that I was kind of trapped, because I wasn’t allowed to go to work, and it was a bit misleading because the pregnancy itself wasn’t a problem, but it was more the circumstance...

Participant 4, focus group 1

I have a background of fatigue syndrome [a Sweden-specific diagnosis equivalent to burnout] and a neuropsychiatric disorder, ADHD combined, which makes my mood automatically fluctuate and maybe is worsened in certain situations in life, just like childbirth...the research is not adapted to people with for example anxiety problems or a neuropsychiatric diagnosis, and then it becomes misleading in the research because it shows that I’m suffering from for example depression, although I’m not.

Participant 40, focus group 13

When informed that the research team accounts for extraneous factors that impact their mood, users reiterated their desire to know more about how the data are used. Issues with accurate tracking of physical activity patterns were another concern for users. In general, participants’ uncertainties about the quality of their data led to hesitations about continuing participation.

...you get a little worried [that you don’t actually contribute] if you think that “I’m collecting a lot of data here, but [I] don’t feel that it might be right.”

Participant 27, focus group 9

### Motivators and Incentives

Motivators and incentives refer to factors that drive users to join the study and continue participating.

#### Contributing to Research

Participants unanimously described their initial motivation to download the app being the desire to contribute to research, particularly on women’s health. Participants felt confident in the value and credibility of the findings that would result from their participation, which also made them feel good about themselves.

The reason why I downloaded the app was precisely to answer these research questions and to be part of the research study itself, so for me it was just to sort of answer the questions.

Participant 1, focus group 1

[This app] is not just trying to sell us products and buy more, but this really has value on a higher level, which hopefully can help others.

Participant 33, focus group 11

I have a sister who got postpartum depression, and so I thought it was kind of good to be part of a study like that and to keep an eye on yourself as well.

Participant 9, focus group 2

#### Advantages of App-Based Research

Since the app is continuously present on the phone and sends notifications when new surveys arrive, participants reflected on how that afforded them flexibility and convenience, especially for new mothers or participants with other children. They found it less effortful than submitting data by other means of collection, such as email, paper, or in-person surveys.

It feels more accessible than getting a link in an email that you have to open. But the surveys are there when you open the app, and then there is a reminder that “you have a survey to answer in the app.”

Participant 29, focus group 10

It’s nice to be able to answer when you can and do it from home, and not have to set aside so much time each time, but you can sort of start and then pause if you don’t get it done and then it stays.

Participant 36, focus group 12

Furthermore, participants reported feeling more comfortable and answering more honestly on PND questionnaires when done on the app versus in person with the midwife.

[My midwife] is not really judgmental, but...I would find it difficult to answer anything other than very positively to a survey that you fill in while someone is staring at you.

Participant 6, focus group 2

#### Personal Health Benefits

One participant described the experience as an “information exchange,” as users benefitted from both general and personalized information and support for their well-being in exchange for providing data. Participants valued getting a statistical overview of their mood and activity patterns based on their data over time, as it enabled them to reflect on their well-being and how to improve it. It incentivized participants to respond to surveys more seriously, knowing that it helps them as much as it would help the research team.

It has been valuable to me both during the pregnancy and after because I’ve had tendencies towards postpartum depression, and I was also a bit vulnerable before the birth...it has been interesting to be able to see [your statistics] and use it in your self-analysis...

Participant 12, focus group 3

It was very clear to me how [my mood] was connected with sleep and so on, then it felt easy, getting it so black and white, it made it easier to sort of plan or prioritize...and keep an eye on my mood a bit.

Participant 14, focus group 4

Furthermore, answering questions about mood prompted users to reflect on their mental well-being and check in with themselves regularly. It was particularly constructive for new mothers and participants with other children to be reminded to self-reflect. In fact, some participants disliked that these surveys became less frequent after birth and would have liked to continue answering them regularly. Moreover, seeing surveys concerning mental health helped normalize and reduce the stigma surrounding poor well-being. Participants felt that “because [the researchers] ask this, there must be others too” (participant 33, focus group 11), and just having the app made them feel less alone through their perinatal journey.

Most of all, users valued the notification they received when their scores on the EPDS were high, as it forced them to acknowledge and take their symptoms seriously and to consider seeking help.

I don’t think I understood myself that I felt as bad as I really did...so for me it has been the absolute best thing about this app that you get detected, so there was still a purpose to follow how you feel...

Participant 21, focus group 7

I was diagnosed with depression during pregnancy...even starting to seek treatment for it at all, it was a combination of the app signaling it, and then you were given the opportunity to talk to someone on the app...the person I spoke to on the Mom2B app said “get in touch with your midwife because you’re not feeling well,” so that led me to seek care...

Participant 39, focus group 13

However, some participants felt that the 5-point scale for the well-being surveys was unable to capture the nuances in their moods, and as such, they felt they incorrectly received notifications to seek help.

In addition, participants found the weekly information reports fun to read and educational and described them as “reliable” and “factual.” However, there was a general dissatisfaction with their conciseness, and desire for them to be more detailed and informative. As such, users did not consider Mom2B as their primary source of general perinatal information but would have preferred to do so to “have everything in one place,” especially since they trusted it more than a commercial app. It also allowed participants to keep track of what perinatal week they are in, which was otherwise confusing for some. Some participants would have liked to get more practical tips and advice from the reports and relevant content for those having multiple pregnancies.

It’s not as comprehensive as a lot of other apps are, so if I want to know something about the baby’s development, or what’s happening in that week of pregnancy, I’ll go to some other app.

Participant 31, focus group 10

### Barriers to Task Completion

The completeness of data collected is a vital characteristic of its quality. Mom2B participants had to complete surveys and voice recordings regularly, and our results highlight 4 main reasons that hindered them from doing so.

#### Unwillingness to Share Data

Women described abandoning surveys due to either not wanting to answer or not knowing or remembering information. The majority of responses were related to recording weight, and it was clear that women found the task of weighing themselves distressful and wished to avoid it.

I don’t know my weight, and don’t want to know my weight, it’s not good for me to know my weight, and then I can’t answer them...it would have been nice to just write “I don’t know” instead of not being able to answer that survey...

Participant 14, focus group 4

#### Practical Barriers

Participants found it difficult to complete voice recording tasks, as they struggled to find the time or a quiet environment. This was especially the case for women with a newborn infant or with other young children at home and was exacerbated by the stay-at-home policies imposed due to the COVID-19 pandemic.

#### Unclear Instructions

Participants experienced confusion with completing certain tasks that they felt lacked clear instructions. One participant described uncertainty in how fast to speak or what tone to use when recording voice. Another described how it “wasn’t entirely clear when to use periods or commas when entering weight” (participant 26, focus group 9).

With frequently recurring surveys in particular, such as the weekly well-being checks, some participants reported the monotony of the questions inhibited them from reflecting on how they really felt.

I really tried to stop and think “how am I really feeling? How has the last week been?” it was a great way to pause, but I’m afraid that somewhere subconsciously I still answered a little habitually...a week goes by so fast, so it feels as if you have just answered them.

Participant 15, focus group 4

#### Task Load

Participants agreed that the number of surveys they need to complete in any given week can feel overwhelming and daunting. One participant reported feeling “constantly behind.” On the other hand, participants also found the individual surveys to be short and easy to answer and appreciated that the surveys were categorized by priority so they knew what is most important to answer.

### User Experience

Users discussed usability, customizability, and accessibility as impactful determinants of their user experience.

#### Usability

Usability refers to how easily and frictionlessly the user interacts with the app and uses its features. For the most part, participants described the app as user-friendly and “easy to understand.” Although some felt the interface was a little unsophisticated and boring, others felt that its simplicity made the app feel secure and serious.

I don’t feel that the app is really bad, but when time, money and resources are available, you can improve it.

Participant 21, focus group 7

Participants often felt an inadequacy of guidance and information in the app for performing tasks and resolving common issues. Uncertainty about the length and expiry time of tasks often led to hesitations to start the task or to miss it.

...it’s good to have some kind of time indication...so that you can think “okay, I have two days, I might not be able to do it right now, but I’ll still try to do it within two days.”

Participant 1, focus group 1

Insufficient information may have also affected the discoverability of features and content in the app, as several users were unaware they could adjust their labor date, view statistics based on their data, and continue the study after birth or if they had a second child.

There are several features in the app that I only noticed now that I looked through it a little more closely to be part of this interview, like statistics...in other apps it’s a bit more smooth-flowing, it’s hard to miss functions.

Participant 6, focus group 2

Participants would have liked to have more and clearer information available related to resolving technical issues and frequently experienced problems to avoid the inconvenience of waiting for email responses from the support team. One participant described switching to a new phone and being unable to log back into the app for 2 months while waiting for a response from technical support:

There are quite a lot of people who change their phones often, so it’s a very unnecessary omission [of information] when it’s so common.

Participant 20, focus group 6

Technical issues were a major source of friction, and it was apparent that they affected participants’ motivations to continue, especially when the issue was related to providing data. Social media and movement data were not recorded correctly for most participants, which lowered the incentivizing impact of the statistics graphs. Users often had difficulties logging back into their accounts, for example, if they switched to a new phone, and found that their previous activity had not been saved on the app.

I was in contact with you [the support team], and you said that “just ignore that [tasks] you have already answered, because that data is sent in, just continue to answer the new ones that come in.” But then there was so much now, and it wasn’t really possible to tell them apart.

Participant 36, focus group 12

Other issues participants described as frustrating were difficulties logging in, app draining battery or crashing, and glitches with surveys. Some participants experienced incorrectly occurring notifications to answer surveys; however, most participants were content with the frequency of notifications and considered them necessary reminders.

#### Customizability

Customizability refers to enabling the users to personalize the app according to their needs and priorities. Participants with multiple pregnancies or health conditions wished for information from the app that felt more relevant to them. Participants also wanted the option to connect the app with smartwatches or pedometers to track movement better. The task of recording weight was dividing in particular. Most found it undesirable and expressed the need for opt-out response options such as “Prefer not to answer” so that they could remove such surveys and also not have to simply abandon them. Others wanted to track it more often and proposed being able to record weight manually as a solution.

In order for it not to be triggering and if you yourself want more statistics, you could make it so that you could add them more often yourself.

Participant 12, focus group 3

I felt like “God, I can’t even send [the survey] away...and there isn’t an alternative”...

Participant 32, focus group 10

#### Accessibility

Accessibility refers to how comfortably users with different needs and abilities are able to use the app and its features. One participant described the font as being too small to read comfortably. Two others commented on the complexity of the text for people with reading disabilities and suggested having the option to choose simplified Swedish.

You [should be able to] choose whether you want simplified text or not, because there are a lot of people who have hidden dyslexia, and may not understand all the concepts.

Participant 40, focus group 13

One participant also noted how maternity clothes often lack pockets to carry one’s phone in, which makes it problematic to share movement data:

even if I had [been physically active], or I have a job where I stand and walk a lot at times, it kind of didn’t show up in the app at all because the phone was on a bench.

Participant 28, focus group 9

## Discussion

### Principal Findings

App-based digital phenotyping is a rapidly growing method in health care research with little to no studies evaluating user experience and the barriers and facilitators of user engagement [37]. This study explored pregnant and postpartum women’s views and experiences with the Mom2B app, including how they perceived various features, and the factors that impacted their continued use of the app. Overall, participants deemed app-based digital phenotyping as an acceptable and feasible method of sharing data for research, especially longitudinal research, as it afforded them convenience and flexibility while also allowing them to benefit personally from the data they share by monitoring their well-being. Our findings highlight a duality in how the Mom2B app is perceived by users as both a tool for research and an mHealth app. While data collection for research is the primary function of the app and plays a bigger role in the initial acquisition of users, the health features are what motivate the long-term retention and continued engagement of users, which are essential for minimizing the risk of missing data.

It is important to consider the cultural context of this study, being focused on the Swedish population. Sweden, like other Nordic countries, has one of the highest rates of smartphone penetration in the world within all age groups as well as high rates of digital health care practices among the general population [8]. This makes smartphone-based digital phenotyping exceptionally efficient in this population due to the commonplaceness of the technology and its use in health care activities. Trust and engagement in research as well as openness to technological developments and use also make the Swedish population uniquely easy to implement such technologies with [38], although the barriers and challenges to usability and continued engagement are not very different from other populations.

Transparency, as a characteristic of digital phenotyping research, was valued by all participants. Participants evaluated this research positively for transparency but expressed their desire to better understand why each type of data is needed and how exactly it is being used in the research, as it related directly to their willingness to consent to sharing different types of personal data. Participants also appreciated the control they have over deciding which type of data they want to share or not, which is consistent with studies showing that users prefer dynamic and flexible consent models that give them more control [39].

These findings also aligned with previous research [40,41], showing that participants felt more willing to share data with and use an app that was developed by university-affiliated researchers, as it led to better expectations of protection of their data in comparison with commercially developed apps. Moreover, despite concerns regarding the sensitive and personal nature of data requested from participants, studies have shown that they are generally motivated to consent to sharing data for the purpose of research and improving health care provision [24,39]. In fact, the majority of the participants in this study were motivated primarily by the desire to support the research effort and possibly help other women. While these findings agree with previous studies done in various countries, it is also important to note here the exceptionally high public trust and commitment to research in Sweden. A 2022 report by the Swedish nonprofit organization, Vetenskap & Allmänhet (Public & Science), shows that 89% of women in Sweden have high confidence in researchers and universities and believe it is important to be involved in research [38]. However, for continued engagement with the app, more direct and personal incentives are important for users [20]. Three features were particularly incentivizing for our participants to continue sharing data and engaging with the app: the statistics and the high EPDS score alert, which enabled users to self-monitor their well-being throughout the perinatal period, as well as the weekly reports, which participants found enjoyable, interesting, and educational.

Fundamentally, it appears that women are motivated by a sense of social responsibility, concern for their health, and curiosity and interest. Intrinsically motivated behaviors have been described in the literature as generating persistence and long-term stability in behavior [20,42], which is especially valuable in longitudinal studies like Mom2B. Furthermore, self-monitoring mechanisms in mHealth apps have been shown to motivate long-term use of such tools because of the value of understanding one’s own psychological well-being [20,24,43]. These findings emphasize the importance of designing features that provide clear personal benefits to the user to increase the perceived use of the app. Considering the perceived duality of this app, it is important to keep in mind that although the primary function of this app is to conduct research and acquire data from users, it is the personal benefits they get from the app that largely motivate them to share data and engage with the app over time. Engagement with the app is needed for the continuous collection of passive data, as long periods of inactivity can compromise or stop passive data collection altogether [44].

Participants in this study offered several suggestions on how the Mom2B app could be improved, as the general preference was to have a single app from a trustworthy source that met all expectations in terms of features, instead of having to use other commercially developed apps that participants considered less reliable. Weekly information reports should be at least on par with commercial apps in terms of the detail and length of information and be customizable to women experiencing multiple pregnancies. Customizability of the app is an important area of improvement, as giving users a sense of control over the app directly impacts the perceived ease of use, efficiency, and user satisfaction [29,41,45]. One feature users wanted more control over was weight tracking, which received mixed reactions. Enabling users to additionally input weight manually would amplify interaction and engagement from those who wish to track it more often. On the other hand, for those who find it undesirable to track weight, enabling them to skip weight questions would minimize frustration and perceived task load due to unwanted lingering surveys. In general, task load and survey repetition should be carefully determined in mobile research apps, as too many surveys accumulating after brief periods of inactivity were overwhelming for participants and deterred participation. Giving users alternative response options that allow them to skip certain sensitive surveys and remove them from their task list can reduce the perceived task load and improve user experience.

Notifications were an important feature for participants and were evaluated as sufficient and facilitative. Participants were only notified when new surveys or weekly reports became available in the app, which was quite frequent. As such, the Mom2B app does not send reminder notifications, as they may pose a risk of being considered bothersome. Finding the right balance for notification frequency can be complicated, which is an important reason for customizability and is enabling users to alter notification preferences within the app [29,46]. Another issue is that of technical problems and system errors, which can decrease the perceived ease of use and the motivation to continue using the app. It is important for users that system errors are appropriately explained and that a solution is available without much effort or that support for technical issues is easy to access and resolves the issue quickly [29].

According to our findings, most participants found it difficult to make voice recordings after birth when the infant’s needs and frequent crying can be a barrier to record. Implementing accessible designs is especially necessary for user groups such as women in the perinatal period, due to the various barriers and limitations they may experience. Pregnancy clothing often not having any pockets is another limitation experienced by participants, preventing them from accurately recording and tracking their mobility. Designing for inclusivity can be facilitated by testing the designs with users or including them in the design process. Our findings emphasize the importance of user testing of the app in an early stage, as it would also refine the overall usability and improve the acceptability of the app and the study.

### Strengths and Limitations

This study included a large number of women interviewed about their perspectives on the Mom2B app as users. We made an effort to recruit a participant group that was representative of women who had used the app in both the pregnancy and the postpartum period as well as both those who had and had not experienced symptoms of depression while using the app. This was done to ensure we captured user perspectives and experiences reflecting the full scope of app features, some of which may not have been available, for example, for those who did not display depression symptoms or did not participate during pregnancy, as well as to ensure that women displaying depressive symptoms were sufficiently represented. Not surprisingly, women with experiences of depression were a relatively small group due to a higher rate of participation cancellation; however, purposive sampling may have prevented this group from completely being lost to attrition.

Furthermore, although focus group interviews are traditionally conducted in person, findings from recent studies substantiate that output, engagement, and participant satisfaction are not affected by engaging remotely [47,48]. In fact, remote interviews were especially suited for our population, as they allowed us to recruit from a more diverse pool of participants in Sweden and build a more representative sample. Face-to-face participation would have been challenging for our participants, as most were either in the late stages of pregnancy or newly delivered mothers, and the inconvenience of unnecessarily traveling even short distances would have likely led to far more dropouts.

Ultimately, the number of participants in most focus groups was still less than what is generally considered the ideal (5-8 participants) [32]. However, this was observed to be advantageous for this particular group, as it afforded each participant more time to share and discuss their experiences given their limited availability. Nevertheless, it is possible that smaller focus groups may have been deprived of achieving the same quality of discussion as larger groups. One focus group interview turned into an individual interview due to other participants dropping out, which resulted in a lack of group discussion. We decided to include this interview anyway, as the participant shared unique insights on their experience that we considered important.

Moreover, since an open invitation to participate was sent to Mom2B participants, we considered the possibility that the participants may have predominantly been users who are more technologically savvy and frequent users; however, based on our conversations with interview participants, this did not seem to be the case. Interviews were conducted by a female research assistant, which was considered important to allow the participating women to feel at ease and reduce participant bias.

One important limitation to consider is the lack of usability testing in this study. Having participants actively use and explore the Mom2B app during the interview as well as giving them tasks to perform such as answering a survey, checking their monthly activity, or reading the recent weekly report may have enhanced the detail and specificity of the feedback they provided as well as triggered memories of past experiences. Future studies are encouraged to use usability testing in conjunction with focus group interviewing when exploring user experiences of such apps.

### Conclusions

This study adds to the limited literature examining user experiences and attitudes toward digital phenotyping apps in the area of mental health research, particularly in the perinatal period. Participants shared their insights on barriers and facilitators of app use and study participation as well as suggestions for the improvement of features and user experience. These results serve as a foundation for app developers and health care researchers in creating apps for research and contribute to our understanding of the opportunities and challenges in designing and implementing apps to support longitudinal research using digital phenotyping.

## Appendix

Multimedia Appendix 1 Interview guide (English translation).

## Abbreviations

COREQ: Consolidated Criteria for Reporting Qualitative Studies
EPDS: Edinburgh Postnatal Depression Scale
mHealth: mobile health
PND: perinatal depression

## Data Availability

The data sets generated and analyzed during this study are available from the corresponding author on reasonable request.
