# Supplementary material for: Exploring User Experiences of the Mom2B mHealth Research App During the Perinatal Period: Qualitative Study
Source: JMIR Form Res. 2024 Aug 8;8:e53508. doi: 10.2196/53508 (PMC11342009; doi:10.2196/53508)
Supplement: Multimedia Appendix 1 [file formative_v8i1e53508_app1.docx]

PART 1

Use of the app

1. How long have you been using the app?
2. During which weeks of pregnancy and which weeks with the baby?
3. Tell us about how you used the app? Which features of the app were most useful, such as weekly reports, statistics, journal? Have you deleted the app? How actively did you use it?
4. Tell us about how you used the app? How did you experience the consent issues, for example about location services?
   1. What did you think about giving consent over multiple pages?
5. Is there anything that has not emerged that you would like to add?

PART 2

Attitudes towards the Mom2B app

1. What was the best thing about the app?
2. Does the app have something that other apps don't have that was good?
3. How were notifications perceived? Questions? Statistics? The weekly reports?
4. What was the worst thing about the app?
5. What would you like to change with the app?
6. Is there anything that has not emerged that you would like to add?

PART 3

Research and support for those who feel unwell

1. What are your thoughts on conducting research in this way?
2. What are your thoughts on whether this can be helpful for women who are depressed?
3. Is there anything that has not emerged that you would like to add?

PART 4

Conclusion

1. (The interview facilitator summarises the findings – if necessary, asks for comments and clarifications).
2. Is there anything that has not come to light that you would like to add?
